# Attitudes Toward the Environment and Use of Information and Communication Technologies to Address Environmental Health Risks in Marginalized Communities: Prospective Cohort Study

**DOI:** 10.2196/24671

**Published:** 2021-09-23

**Authors:** Jose G Perez-Ramos, Scott McIntosh, Emily S Barrett, Carmen M Velez Vega, Timothy D Dye

**Affiliations:** 1 Department of Obstetrics and Gynecology School of Medicine and Dentistry University of Rochester Rochester, NY United States; 2 Department of Public Health Sciences School of Medicine and Dentistry University of Rochester Rochester, NY United States; 3 Environmental and Occupational Health Sciences Institute Rutgers School of Public Health Rutgers University Piscataway Township, NJ United States; 4 Escuela Graduada de Salud Pública Recinto de Ciencias Médicas Universidad de Puerto Rico San Juan Puerto Rico

**Keywords:** community engagement, environmental health risk, epidemiology, ICT, mHealth, mobile phone, Puerto Rico

## Abstract

**Background:**

Information and communication technologies, including mobile health (mHealth), can help isolated communities address environmental health challenges. The Puerto Rican island of Culebra has faced multiple sociopolitical and economic factors that have distressed the island’s environment and health. *Culebrenses* are technologically engaged and have demonstrated a use of technology that transcends socioeconomic barriers. As a result, technological interventions could potentially help manage environmental risks on the island.

**Objective:**

This study aims to test and evaluate the potential benefits of an mHealth tool, termed ¡mZAP! (*Zonas, Acción y Protección*), for engaging communities with environmental risks through technology.

**Methods:**

Participants using ¡mZAP! (N=111) were surveyed. Bivariate analyses were used to examine associations of mHealth use with sociodemographics, technology use, an adapted environmental attitudes inventory, and the multidimensional health locus of control. Logistic regression was used to examine associations between attitudes toward environmental health risks and mHealth use.

**Results:**

Higher positive attitudes toward the environment were significantly associated with the use of ¡mZAP! (odds ratio 5.3, 95% CI 1.6-17.0). Environmental attitudes were also associated with the multidimensional health locus of control *powerful others* subscale (*P*=.02), indicating that attitudes toward the environment become more negative as feelings controlled by others increase. Participants felt that the authorities would resolve the challenges (63/111, 56.7%).

**Conclusions:**

Perceived lack of control could present barriers to collective actions to address salient environmental health challenges in communities. The ongoing dependency on government-based solutions to community problems is worrisome, especially after the hurricane experiences of 2017 (which may potentially continue to be an issue subsequent to the more recent 2020 earthquakes).

## Introduction

### Background

Information and communication technologies (ICTs), including mobile health (mHealth), can help isolated communities address environmental health challenges [1,2]. The World Health Organization considers environmental risks a public health priority [3]. Social and ecological determinants of health, along with environmental health challenges, often exacerbate pre-existing health disparities [4]. Traditional health paradigms have been changed by mHealth tools to address such health disparities. For example, a study used a mobile app termed *FAITH!* (Fostering African American Improvement in Total Health) to improve cardiovascular health among an African American church community (n=86) in the United States following a multiphase community-based participatory research design [5]. Other examples include using a web-based intervention for smoking cessation among low socioeconomic status populations in the United States (n=1440) [6] and an mHealth tool to reduce health care access gaps among marginalized rural populations in Laos (n=983) and Thailand (n=1158) [7].

The benefits of using ICTs to address environmental risks following crowdsourcing approaches have been successfully implemented in the United States using the mobile phones of users [8,9]. Mobile phones are also being used to engage populations in new and innovative learning modalities [10,11]. Previous studies have shown that mobile tools such as cell phones can help users develop positive attitudes toward the environment [12,13]. Mobile tools facilitate engaging with users by using their devices (eg, smartphones and smartwatches) as part of the solution to existing environmental challenges [8,13].

Although the use of technology has transcended socioeconomics barriers, providing digital access to different levels of society, a significant gap perpetuates digital access disparities among marginalized communities worldwide [14-16].

The power of being interconnected via modern technology (eg, social media, cell phones, and the internet) has been shown to (1) catalyze sociopolitical changes [17,18], (2) close the gap in physical distances [19], (3) foster the development of health advances [20], and (4) provide access to information [21]. In contrast, the use of technology has also been linked to the spread of misinformation, resulting in the exacerbation of social injustices among marginalized groups. For example, WhatsApp was used in Maceió, Brazil, to diffuse conspiracy theories about vaccines as the cause of microcephaly in children testing positive for the Zika virus [22,23]. Thus, communities often have untapped and unrecognized resources that may be mobilized to work with the government sector, health programs, and nonprofit organizations to address risks in their environments [24].

Many communities, particularly islands, are dependent on ICT to reduce the impact of their geographical and societal isolation [25]. Island communities face many social and health challenges that are not necessarily faced by other communities, which can intensify risk [26-28]. The remote island of Culebra, off the eastern coast of Puerto Rico’s *Big Island* (*Isla Grande*), is geographically and systemically isolated from the rest of Puerto Rico. Culebra’s isolation also results in limited political power because of the small population, leading to a system of dependency that negatively impacts health access, utilities, waste management, transportation, water safety, and air and soil contamination, which is common in rural and isolated areas [29-33].

Hurricanes Irma and María in 2017 demonstrated this dependency, with Culebra losing connection to the national electrical grid for more than a year [34] and having difficulties in resuming ferry and other island services. Although impacted by these challenges, *Culebrenses* have a strong history of collective engagement against social injustices, such as facilitating the US Navy’s exit from the island in 1975 and protesting against maritime transportation policies [35]. In addition to this community engagement, *Culebrenses* are technologically engaged and have demonstrated a use of technology that transcends socioeconomic barriers [36]. As a result, technological interventions could potentially help manage environmental risks on the island.

**Figure 1:**
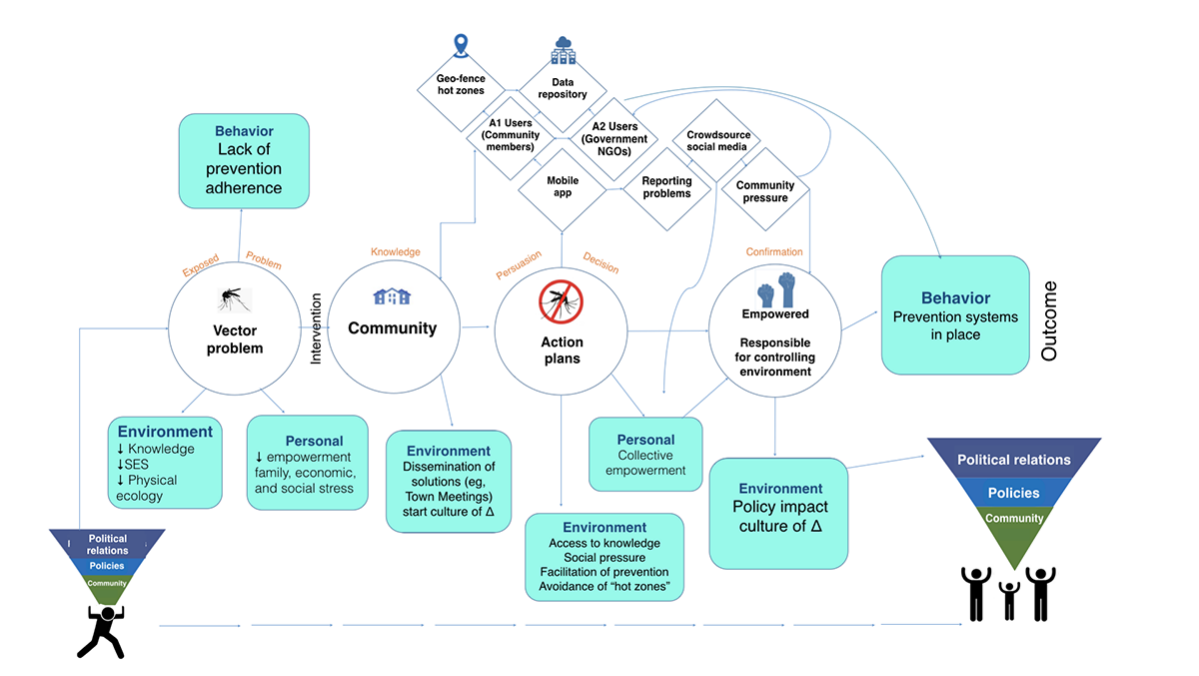
Community-centered environmental health risk control model. NGO: nongovernmental organization; SES: socioeconomic status.

This study was guided by a multi-theoretical framework (social cognitive theory [SCT] and the diffusion of innovations theory) which, along with community-centered design methodology, can (1) provide a model for behavioral changes and (2) guide the process to understand the adoption of this mHealth tool better [37-39]. This study also followed a human-centered design framework that provides an inclusion process whereby users are central and active agents necessary for the conception, testing, implementation, and evaluation of solutions to Culebra’s environmental health risk challenges [40]. The SCT addresses behavior change and the effect of the community members’ environment [41]. The SCT also helps identify community problems and priorities, allowing for an in-depth understanding of personal factors, current community behaviors, and environmental influences associated with environmental health risks that can affect community-wide behavioral change. The diffusion of innovations theory facilitates the process of development and sustainability [42]. Owing to the complexity of these two theories, a theoretical framework model titled *Community-Centered Environmental Health Risk Control Model* was developed for this study (Figure 1).

### Objective

Following this theoretically informed model facilitated the work as part of a community-driven research project to collaborate with community partners to implement a tailored, community-centered, and crowdsourced ICT termed ¡mZAP! (*Zonas, Acción y Protección*) as part of a community-driven approach to assess the ability of ICTs to engage islanders in collective environmental actions. Specifically, in this study, we evaluate the ability of psychosocial, environmental attitudes, and technological variables to predict the use of the ¡mZAP! app.

## Methods

### Overview

We used a prospective design to assess the ¡mZAP! app use. The study surveyed community participants at baseline to capture their perceptions and attitudes toward the environment, access to and use of technology, community actions toward environmental challenges, knowledge about vector-borne diseases, and health control in Culebra, Puerto Rico. App registrants were followed prospectively for 3 months (November 2018-February 2019) to assess the use of ¡mZAP!

### The Archipelago of Culebra

**Figure 2:**
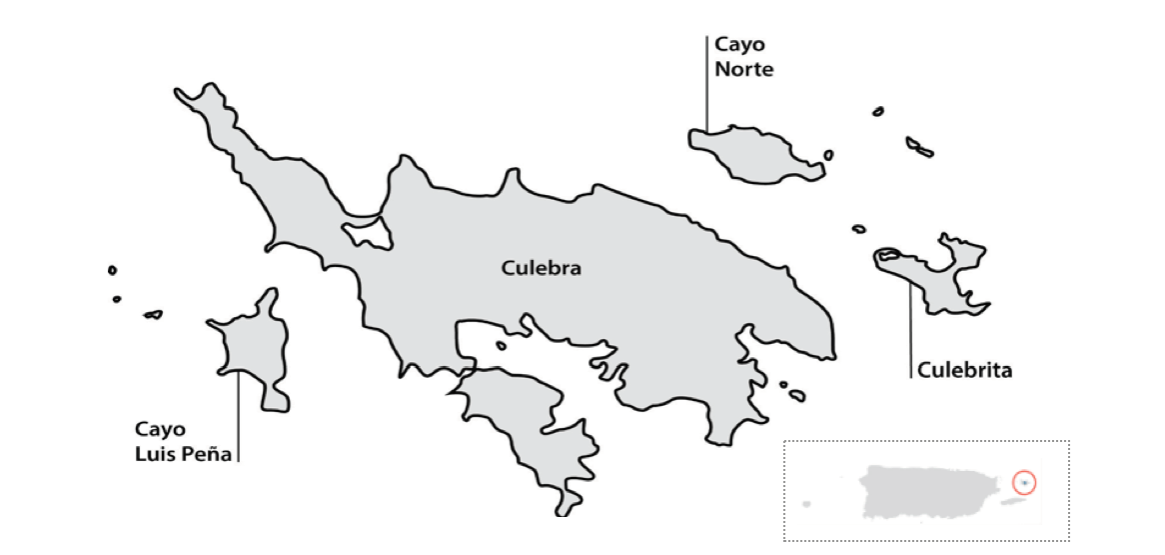
Map of Culebra in relation to the rest of Puerto Rico.

Culebra is an archipelago municipality located on the east coast of Puerto Rico, 17 miles from *Isla Grande* (*Big Island*—as it is commonly referred to in Puerto Rico by the residents of outer islands; Figure 2). On the basis of the 2018 US Census annual estimates of the resident population, Culebra has an estimated population of 1500 [43]. Culebra’s geographical location provides a unique, biodiverse ecosystem of drylands, mangroves, lagoons, white sand beaches, and coral reefs, all in almost 12 square miles [35,44]. Largely owing to this biodiversity and geographical location, tourism is Culebra’s primary economy.

Culebra also presents another face; for many decades, this set of islands has experienced marginalization by the United States and from the local government [33,35], including military bombing drills that left unknown contamination and pollution effects [29-31], unreliable maritime transportation [45], environmental hazards [46], high presence of vector agents (mosquitoes) [35], and the lack of a proper health care facility [32,47].

### ¡mZAP! App

The ¡mZAP! app was designed and developed as a community-oriented ICT to stimulate collective action to mitigate environmental health risks (eg, trash and mosquitoes). This study design involved direct input from community organizations and community members in Culebra, including women’s advocacy organizations, sports organizations, private businesses, health organizations, and schools, primarily through focus group and qualitative engagement processes, as previously described [48].

In this app, users may take actions to identify, report, and address community-based priorities such as abandoned structures, trash, stray animals, stagnant water, mosquito breeding grounds, and pests. Although not directly relevant to environmental issues, a feature to track ferry and ship locations was included to further incentivize app use as the community had identified maritime transportation as a major challenge.

### Survey

A survey was deployed at baseline using the REDCap (Research Electronic Data Capture, Vanderbilt University) platform version 9.0, which also hosted the survey data. As defined in previous studies, the REDCap database is a secured, encrypted, and Health Insurance Portability and Accountability Act–compliant data capture application developed for large-scale research projects [49].

The survey contained adaptations of two previously validated instruments and original items self-administered by participants via the mobile app tool. The survey aimed to better understand users’ attitudes toward the environment, community technology use, and community challenges. Questions related to community health, demographic characteristics, the environment, attitudes toward community actions, and technology use were included. Consistent with recommendations for pretesting and pilot-testing of surveys evaluating mHealth interventions [49,50], the survey was pretested with approximately 10 iterations with feedback from 4 bilingual Latinos from the author’s institution and volunteers from the target community.

### ¡mZAP! Databases

The ¡mZAP! app was built as a cross-platform mobile and web-based app using the *Ionic* framework by an app developer that worked as part of the ¡mZAP! core team at the University of Rochester. *Ionic* is an open-source toolkit that facilitates the building of mobile apps and web-based applications using a single coding structure (eg, HTML, Cascading Style Sheets, and JavaScript), eliminating the need to create a new coding process for each mobile and web-based platform. An integration of *Angular*, a Google-maintained platform, was used to build the app across mobile and web-based systems (eg, iOS and Android). *Firebase* software was used as a database repository. *Firebase* is a Google-based software with the capacity to secure file uploads and downloads, including images, videos, and other user-generated data under Google encrypted (AES256 and AES128) and cloud storage. The ¡mZAP! mHealth tool back-end process was built to assign each user a unique ID linking all the data generated by users while using the app. This process facilitated a better understanding of ¡mZAP! users’ behaviors. An application programming interface was used to access the REDCap survey directly from the app. Unique user identifications and email addresses were used to merge the two databases (¡mZAP! use and the REDCap survey). A weekly data transfer stored participants’ app use to secure servers at the lead author’s institution.

### Sample Size Calculation

This study was powered to detect an effect size of 3.0 (α=.05 and β=.20) of app use by the environmental attitudes’ category. We estimated that 50% of participants with positive attitudes toward the environment would use the app and 25% of participants with negative attitudes would use ¡mZAP! On the basis of these calculations, the sample size required for this study was 116 participants.

### Participants and Study Implementation

Eligible participants were ≥18 years and self-reported living in Culebra for at least 6 months before the start of the study. Survey questions were administered in Spanish, which is the primary language of the island.

**Figure 3:**
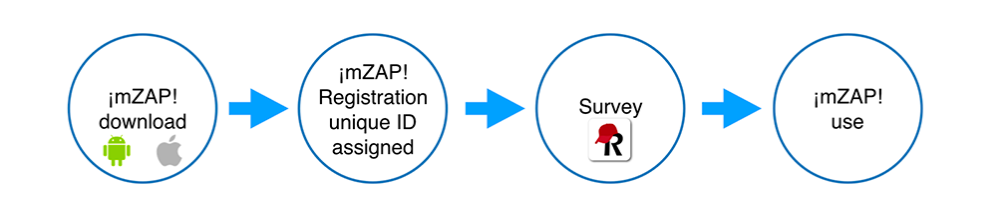
¡mZAP! user implementation process. ¡mZAP!: Zonas, Acción y Protección.

Participants were recruited at baseline following a snowball sampling methodology [51], community meetings, flyer distributions, and social media posts. During in-person visits, eligible participants were invited to download the ¡mZAP! app and complete the survey during the registration process before starting the use of app functionalities (Figure 3). Participants completed the survey using their devices, and via REDCap, the surveys were uploaded to the University of Rochester’s firewall-protected, encrypted cloud-based servers. Participants did not receive any compensation for completing the survey or using ¡mZAP! For recruitment purposes, a local area code telephone number and a unique email address were acquired, and a Facebook fan page was created specifically for the project, enabling participants to reach the research team for questions and concerns about the project. This study adhered to the Reporting of Studies Conducted using Observational Routinely Collected Data guidelines and checklist to ensure the inclusion of all components deemed necessary for a scientific research report [52].

¡mZAP! was launched in a series of in-person community meetings in fall of 2018 and was disseminated among community members during a 3-month trial period (November 2018-February 2019). A total of 124 people downloaded the app (Apple iOS 53/124, 42.7% and Android 71/124, 57.3%). Out of the 124 people, 116 (93.5%) participants started registration; 5 (4%) respondents did not meet the inclusion criteria of being a resident of Culebra and were excluded. Out of the 116 participants, 111 (95.7%) participants successfully registered and completed the survey.

This study was reviewed and approved by the institutional review board of the first author’s (RSRB00073777) university and followed the 1964 Declaration of Helsinki and its subsequent amendments. HealthproMed, a federally qualified health center in Culebra, reviewed the study protocol and approved the study. An information letter was electronically provided to the participants during their registration for the app.

### Measures

#### Overview

The study data included two distinct databases: ongoing baseline survey data and ¡mZAP! use data. The app documented user actions and collected data within a 3-month period. *¡mZAP! use* was defined as any action by users within the mobile app (eg, reports, comments, messages, likes, and resolves) during this follow-up period. Multiple user data points were synchronized by random user unique identification, which traced each user’s behavior while using the ¡mZAP! tool.

#### The Environmental Attitudes Inventory

The environmental attitudes inventory [53] scale was adapted (adapted environmental attitudes inventory [a-EAI]) to assess participants’ attitudes toward the environment. This environmental scale was developed to evaluate the multidimensional nature of environmental attitudes and the perceptions or beliefs of participants regarding the natural environment and associated factors. For this study, 12 items from the environmental attitudes inventory scale were selected and combined for the a-EAI summary scale. We converted the EAI summary scale to a standardized *t* score with mean 50 (SD 10) [54]. In addition to analyzing the a-EAI as a continuous variable, we reduced the a-EAI to discrete categories with tertiles as the cutoff.

#### The Wallston Multidimensional Health Locus of Control

The multidimensional health locus of control (MHLC) scale assesses perceptions of control over one’s health. The MHLC scale has been used in previous studies to measure the influences and perceptions of community members regarding external factors that affect health outcomes [55]. A Spanish-validated version of the MHLC was used in this study [56]. The three subscales were MHLC internal (internal health locus of control), MHLC external (powerful others health locus of control), and MHLC chance (chance health locus of control) [57]. The rationale for using the MHLC was that perceptions of health control could be associated with overall control and a positive inclination to act against environmental health risks. The subscale scores were standardized using the Streiner and Norman [54] method.

#### Community Environmental Priorities, Technology Use, Mosquito Knowledge, and Demographics

For addressing community environmental priorities, two additional sections were incorporated from previous study findings [48,49,58] to assess the use of technology, environmental challenges, community resolutions, and access to technology at home (eg, cell phones and the internet).

### Data Analysis

The survey data were exported from REDCap, cleaned, and analyzed using SPSS Statistics (IBM Corporation) version 25 [59]. Chi-square tests and analysis of variance were used to test bivariate associations among all key variables. Odds ratios (ORs) with 95% CIs were used to examine the magnitude of the association of predictor variables, including the main predictor variable (attitudes toward the environment, a-EAI) and the primary outcome (use of ¡mZAP!). Forced and forward stepwise logistic regression models [60] were generated to control for potential confounders that were marginally associated (ie, *P*<.25) with a-EAI and mZAP! use. Where necessary, *P*<.05 was considered significant.

## Results

### Study Sociodemographic Characteristics

In total, 110 participants completed the download of ¡mZAP! and the intake survey. As Table 1 shows, most (71/110, 64.5%) of the survey participants were aged >35 years. In addition, 65.4% (72/110) had completed a higher education degree and 68.5% (76/111) had lived in Culebra for >11 years. An equal number of females and males participated in the study (n=54 each). A comparison of the study sample with the US Census 2013-2017 American Community Survey in Puerto Rico [43] revealed no statistically significant differences between the study population and the population of the municipality of Culebra regarding gender and ethnicity (data not shown), suggesting that the study sample was similar to the overall population of Culebra.

**Table 1:** Sociodemographics and technology attitudes among ¡mZAP! (Zonas, Acción y Protección) study survey participants in Culebra, Puerto Rico, 2018-2019 (N=110).

Variables		Participants, n (%)
**Age (years)**		
	18-25	10 (9.1)
	26-36	29 (26.4)
	37-47	34 (30.9)
	48-54	22 (20)
	≥55	15 (13.6)
**Age category (years)**		
	≤36	39 (35.5)
	≥37	71 (64.5)
**Education level**		
	Less than or equal to high school	34 (32.1)
	Higher education	72 (67.9)
**Gender**		
	Female	54 (49.1)
	Male	54 (49.1)
	Prefer not to answer	2 (1.8)
**Time living in Culebra (years; N=111)**		
	1-10	35 (31.5)
	More than 11	76 (68.5)
**Do you have a cell phone data plan?**		
	Yes	105 (96.3)
	No	4 (3.7)
**How is your cell phone reception on the island?**		
	Good	11 (10)
	Regular or bad	99 (90)
**How many cell phones do you have at home?**		
	1	28 (25.5)
	>1	82 (74.6)
**Does your home have internet access?**		
	Yes	96 (87.3)
	No	14 (12.7)
**What type of internet do you have in your home?**		
	Wi-Fi and satellite	31 (32.3)
	Data plan	63 (65.6)
**How many hours per day do you spend using electronic devices?**		
	<1-5	89 (80.9)
	≥6	21 (19.1)
**Do you use social media?**		
	Yes	105 (95.5)
	No	5 (4.5)
**Daily use of social media**		
	WhatsApp	99 (94.3)
	Facebook	89 (84.8)
	Instagram	43 (41.3)
**Agreement with the following statements**		
	Technology is a tool to help us daily	100 (90.9)
	The use of technologies helps to fix community problems	66 (60)
	The use of technologies brings us closer to the community	37 (33.9)
	The constant use of technology limits the community work	43 (39.1)
	The technology cause problems in the community	36 (32.7)

### Technology and Community Characteristics Among ¡mZAP! Survey Participants

As Table 1 shows, every participant reported owning a cell phone, and 96.3% (105/110) of participants also had a data plan as part of their cell phone plan. With respect to technology at home, 87.3% (96/110) of participants reported having internet access at home, with 66% (63/95) using cell phone data plans for connecting to the internet. Most of the participants owned more than one cell phone (62/83, 74.6%), used electronic devices for up to 5 hours daily (89/110, 80.9%), and had negative perceptions about cell phone reception (99/110, 90%). In addition, 95.5% (105/110) of participants reported being social media users, with WhatsApp being the most used daily social media network (99/105, 94.3%), followed by Facebook (89/105, 84.8%) and Instagram (43/105, 40.1%).

In a series of statements about the relationship between technology and the community, 90.9% (100/110) of participants believed that technology is a tool that can be useful daily and 60% (66/110) agreed that technology could facilitate the resolution of problems in the community. Participants disagreed or were undecided about the role of technology in bringing communities closer (72/110, 65.4%), whether using technologies was an impediment for individuals to do community work (67/110, 60.9%), and whether technology caused community problems (74/110, 67.3%).

### Attitudes Toward the Environment and Predictors

On the basis of the responses on the a-EAI scale, participants often disagreed with items reflecting negative statements about protecting the environment. For example, 44.2% (49/116) disagreed with “One of the most important reasons to keep lakes, beaches and rivers clean is so that people have a place to enjoy water sports,” and 46.8% (51/109) disagreed with “Modern science will not be able to solve our environmental problems.” Most participants agreed with or were ambivalent about items that reflected positive inclinations toward the environment; for example, “Protecting the environment is more important than protecting peoples’ jobs” (65/109, 59.6%) and “I would like to join and actively participate in an environmentalist group” (73/110, 66.4%). When comparing a-EAI with sociodemographic variables, as shown in Table 2, having a higher educational level (OR 4.5, 95% CI 1.2-16.3) and having lived fewer total years in Culebra were significantly associated with a stronger positive attitude toward the environment (OR 3.0, 95% CI 1.2-7.6).

**Table 2:** Associations of the a-EAI^a^ with sociodemographics, technology attitudes, and knowledge about mosquito-borne diseases among ¡mZAP! (Zonas, Acción y Protección) study survey participants in Culebra, Puerto Rico, 2018-2019.

Variables		Total participants, n (%)	a-EAI, n (%)		Odds ratio (95% CI)	*P* value^b^
			High (more positive)	Low (less positive)		
**Age (years; n=108)**						
	≤36	37 (34.3)	11 (29.7)	26 (70.3)	1.5 (0.6-3.6)	.41
	≥37	71 (65.7)	16 (22.5)	55 (77.5)	Referent	N/A^c^
**Education level (n=104)**						
	Higher education	71 (68.3)	22 (31)	49 (69)	4.5 (1.2-16.3)	.01
	Less than or equal to high school	33 (31.7)	3 (9.1)	30 (90.9)	Referent	N/A
**Time living in Culebra (years; n=108)**						
	>11	73 (67.6)	13 (17.8)	60 (82.2)	Referent	N/A
	1-10	35 (32.4)	14 (40)	21 (60)	3.0 (1.2-7.6)	.01
**How is your cell phone reception on the island? (n=108)**						
	Regular or bad	97 (89.8)	25 (25.8)	72 (74.2)	1.5 (0.3-7.7)	.58
	Good	11 (10.2)	2 (18.2)	9 (81.8)	Referent	N/A
**How many cell phones do you have at home? (n=108)**						
	>1	80 (74.1)	22 (27.5)	58 (72.5)	1.7 (0.6-5.2)	.31
	1	28 (25.9)	5 (17.9)	23 (82.1)	Referent	N/A
**Does your home have internet access? (n=108)**						
	Yes	95 (87.9)	24 (25.3)	71 (74.7)	1.1 (0.3-4.4)	.86
	No	13 (12.1)	3 (23.1)	10 (76.9)	Referent	N/A
**What type of internet do you have in your home? (n=95)**						
	Wi-Fi and satellite	33 (34.7)	6 (18.2)	27 (81.8)	Referent	N/A
	Data plan	62 (65.3)	18 (29)	44 (71)	1.8 (0.7-5.2)	.24

^a^a-EAI: adapted environmental attitudes inventory.

^b^*P* value of chi-square test.

^c^N/A: not applicable for reference groups.

### Collective Action Strategies

A series of questions on the importance of community actions to address environmental risks were posed. As shown in Table 3, most participants (79/108, 73.1%) believed that environmental problems are not typically resolved among community members. Similarly, 58.3% (63/108) of participants expected the government to resolve environmental problems. Other participants expressed that these problems would be resolved by the community (29/108, 26.9%) or would be resolved without making any effort to fix them (28/108, 25.9%). No statistically significant associations were found among the series of items on community actions and a-EAI scale scores.

**Table 3:** Associations of the a-EAI^a^ with community environmental actions resolutions among ¡mZAP! (Zonas, Acción y Protección) study survey participants in Culebra, Puerto Rico, 2018-2019 (N=108).

How does generally environmental differences or problems get resolved in your community?			Total participants, n (%)		a-EAI, n (%)				Odds ratio (95% CI)		*P* value^b^
					Hi (more positive)		No (more negative)				
**Resolved between each other**											
	No	79 (73.1)		20 (25.3)		59 (74.7)		1.0 (0.4-2.9)		.90	
	Yes	29 (26.9)		7 (24.1)		22 (75.9)		Referent		N/A^c^	
**Go to government authorities (police and mayor’s office)**											
	Yes	63 (58.3)		13 (20.6)		50 (79.4)		Referent		N/A	
	No	45 (41.6)		14 (31.1)		31 (68.9)		1.7 (0.7-4.2)		.22	
**Ignore and *pass the page***											
	No	83 (76.8)		21 (25.3)		62 (74.7)		1.0 (0.4-3.0)		.89	
	Yes	25 (23.1)		6 (24)		19 (76)		Referent		N/A	
**Think it will eventually get resolved**											
	No	82 (75.9)		22 (26.8)		60 (73.2)		1.5 (0.5-4.6)		.44	
	Yes	26 (24)		5 (19.2)		21 (80.8)		Referent		N/A	
**It will get resolved by itself**											
	Yes	28 (25.9)		8 (28.6)		20 (71.4)		1.2 (0.4-3.3)		.61	
	No	80 (74)		19 (23.8)		61 (76.3)		Referent		N/A	

^a^a-EAI: adapted environmental attitudes inventory.

^b^*P* value of chi-square test.

^c^N/A: not applicable for reference groups.

### Multidimensional Health Locus of Control

Most participants reported moderate beliefs about health control, MHLC internal (76/118, 64.4%), MHLC *powerful others* (78/108, 72.2%), and MHLC chance (71/108, 65.7%; data not shown). No statistically significant associations were found between the a-EAI and MHLC subscales. In contrast, when comparing MHLC subscales with the a-EAI as a scale score, as MHLC *powerful others* scores increased, overall a-EAI scores decreased (*P*=.02; data not shown). This association indicates that attitudes toward the environment become more negative as the sense that one’s health is controlled by *powerful others* (eg, parents, doctors, and authorities) increases.

### ¡mZAP! User’s Characteristics and Predictors

Of the 14 participants who used the ¡mZAP! app, 5 (36%) participants used the app more than once; 11 (78%) participants used the app to report environmental health risks, including trash, stray animals, mosquito breeding grounds, abandoned structures, and deposits of stagnant water; and 2 (14%) participants commented on reports. Of the 14 users, 3 (21%) used the tool to report the maritime transportation location (location of the ferries). In addition, of the 14 ¡mZAP! users, 9 (64%) expressed the expectation that the government would resolve the environmental challenges in the community.

Equal proportions of women and men used ¡mZAP! As shown in Table 4, most users (11/14, 79%) were aged >35 years, lived in Culebra for >11 years (10/14, 71%), and had completed higher education (10/14, 71%). No statistically significant differences were found between nonusers and users in terms of sociodemographic variables. Although not significant, several variables had substantial ORs (ORs>1.5): age (OR 2.2, 95% CI 0.5-8.4), educational level (OR 1.6, 95% CI 0.4-6.5), cell phone number per household (OR 2.2, 95% CI 0.5-10.6), household type of internet access (OR 1.8, 95% CI 0.7-5.2), daily hours spent using electronic devices (OR 2.8, 95% CI 0.8-9.3), and *the use of technologies helps to fix community problems* (OR 3.1, 95% CI 0.9-10.0).

**Table 4:** Association of ¡mZAP!^a^ use with sociodemographics, technology attitudes, and knowledge about mosquito-borne diseases among ¡mZAP! study survey participants in Culebra, Puerto Rico, 2018-2019.

Variables			Total participants, n (%)		¡mZAP! use					Odds ratio (95% CI)			*P* value^b^
					Yes		No						
**Age category (years; n=110)**													
	≤36	39 (35.5)		3 (7.7)		36 (92.3)		Referent			N/A^c^		
	≥37	71 (64.5)		11 (15.5)		60 (84.5)		2.2 (0.5-8.4)			.24		
**Education level (n=106)**													
	Higher education	72 (67.9)		10 (13.9)		62 (86.1)		1.6 (0.4-6.5)			.46		
	Less than equal to high school	34 (32.1)		3 (8.8)		31 (91.2)		Referent			N/A		
**Time living in Culebra (years; n=111)**													
	>11	76 (68.5)		10 (13.2)		66 (86.8)		1.1 (0.3-4.0)			.79		
	1-10	35 (31.5)		4 (11.4)		31 (88.6)		Referent			N/A		
**How is your cell phone reception on the island? (n=110)**													
	Regular or bad	99 (90)		14 (14.1)		85 (85.9)		N/A			N/A		
	Good	11 (10)		0 (0)		11 (100)		N/A			N/A		
**How many cell phones do you have at home? (n=110)**													
	>1	82 (74.5)		12 (14.6)		70 (85.4)		2.2 (0.5-10.6)			.30		
	1	28 (25.5)		2 (7.1)		26 (92.9)		Referent			N/A		
**Does your home have internet access? (n=110)**													
	Yes	96 (87.3)		12 (12.5)		84 (87.5)		Referent			N/A		
	No	14 (12.7)		2 (14.3)		12 (85.7)		1.1 (0.2-5.8)			.85		
**What type of internet do you have in your home? (n=95)**													
	Wi-fi and satellite	33 (34.7)		6 (18.2)		27 (81.8)		Referent			N/A		
	Data plan	62 (65.3)		18 (29)		44 (71)		1.8 (0.7-5.2)			.25		
**How many hours per day do you spend using electronic devices? (n=110)**													
	≥6 hours	21 (19.1)		5 (23.8)		16 (76.2)		2.8 (0.8-9.3)			.90		
	<1 hour or up to 5 hours	89 (80.9)		9 (10.1)		80 (89.9)		Referent			N/A		
**a-EAI^d^ scores (n=108)**													
	Higher (top tertile) score and lower (lowest two tertiles) score	N/A		N/A		N/A		N/A			N/A		
	Two-third low score	81 (75)		6 (7.4)		75 (92.6)		Referent			N/A		
	One-third high score	27 (25)		8 (29.6)		19 (70.4)		5.3 (1.6-17.0)			.003		
Uses social media (n=110)			105 (95.5)		14 (13.3)		91 (86.7)		N/A			N/A	
**How often do you use WhatsApp? (n=105)**													
	Less than daily	6 (5.7)		1 (16.7)		5 (83.3)		1.3 (0.1-12.2)			.81		
	Daily	99 (94.3)		13 (13.1)		86 (86.9)		Referent			N/A		
**How often do you use Facebook? (n=105)**													
	Daily	89 (84.8)		12 (13.5)		77 (86.5)		1.1 (0.2-5.4)			.91		
	Less than daily	16 (15.2)		2 (12.5)		14 (87.5)		Referent			N/A		
**How often do you use Instagram? (n=104)**													
	Daily	43 (41.3)		8 (18.6)		35 (81.4)		2.1 (0.7-6.6)			.19		
	Less than daily	61 (58.7)		6 (9.8)		55 (90.2)		Referent			N/A		
**Technology is a tool to help us daily (n=110)**													
	Agree	100 (90.9)		14 (14)		86 (86)		N/A			N/A		
**The use of technologies helps to fix community problems (n=110)**													
	Undecided or disagree	44 (40)		9 (20.5)		35 (79.5)		3.1 (0.9-10.0)			.04		
	Agree	66 (60)		5 (7.6)		61 (92.4)		Referent			N/A		
**The use of technologies brings us closer to the community (n=109)**													
	Undecided or disagree	72 (66.1)		10 (13.9)		62 (86.1)		1.3 (0.3-4.6)			.65		
	Agree	37 (33.9)		4 (10.8)		33 (89.2)		Referent			N/A		
**The constant use of technology limits the community work (n=110)**													
	Undecided or disagree	67 (60.9)		9 (13.4)		58 (86.6)		1.2 (0.3-3.8)			.78		
	Agree	43 (39.1)		5 (11.6)		38 (88.4)		Referent			N/A		
**The technology cause problems in the community (n=110)**													
	Undecided or disagree	74 (67.3)		10 (13.5)		64 (86.5)		1.3 (0.4-4.3)			.72		
	Agree	36 (32.7)		4 (11.1)		32 (88.9)		Referent			N/A		

^a^¡mZAP!: Zonas, Acción y Protección.

^b^*P* value of chi-square test.

^c^N/A: not applicable.

^d^a-EAI: adapted environmental attitudes inventory.

No item from the MHLC was statistically significantly associated with the use of the ¡mZAP! app, including when compared with each subscale’s total score (MHLC subscale 1 [internal health locus of control], MHLC subscale 2 [powerful others health locus of control], and MHLC subscale 3 [chance health locus of control]; data not shown).

Higher positive attitudes toward the environment were significantly associated with using the ¡mZAP! app (OR 5.3, 95% CI 1.6-17.0). To assess potential confounding factors, we selected variables that were marginally associated with a-EAI and the use of the ¡mZAP! app, although none of the variables met the statistical criteria for remaining in the model. Clinically relevant sociodemographic variables (education level and age) were forced into the logistic regression model and showed no confounding between environmental attitudes and the use of ¡mZAP! app.

A statistically significant (*P*<.01) association persisted when comparing the highest tertile of a-EAI versus the other two tertiles and ¡mZAP! app use (adjusted OR 5.4, 95% CI 1.4-20.4). The Hosmer and Lemeshow goodness-of-fit test [60] was not statistically significant (*P*=.89), validating the tested model.

## Discussion

### Principal Findings

This study was followed by the *Community-Centered Environmental Health Risk Control Model*. This theoretically informed model facilitated the process of learning about *Culebrenses’* environmental health priorities and, more importantly, the implementation of their community perspectives in the development of ¡mZAP! app as an mHealth tool tailored to the community and by the community. This new model contributes to our understanding of behaviors that can lead to better ICT use among community members, and this model can be adopted in future studies worldwide.

The main findings of this study are as follows: (1) ¡mZAP! users were five times more likely to have stronger environmental attitudes than ¡mZAP! nonusers; (2) a negative relationship between environmental attitudes and the MHLC *powerful others* was observed; and (3) an expectation that the government will meet the needs of *Culebrenses* was found*.*

The study results also suggest that *Culebrenses* have a strong use of ICT, where most participants spend up to 5 hours daily using electronic devices. In contrast, this study also found important aspects of *Culebrenses’* potential interpretations of and implications for their attitudes toward the environment. For example, participants with a low perception of health control were also more likely to have a less positive attitude toward the environment. This association indicates that attitudes toward the environment become more negative as the sense that one’s health is controlled by *powerful others* increases. Higher scores on the MHLC *powerful others* subscale have been previously demonstrated to affect health outcomes negatively [61,62]. This relationship could also lead people to become disengaged in efforts to protect their environment. People may hold conflicting beliefs because most participants agreed with or were ambivalent about items that reflected positive inclinations toward the environment.

As described in previous studies, *Culebrenses* expressed a feeling of *being forgotten* by the Puerto Rican government, which could partly explain why *Culebrenses* have a sense of pride and ownership with respect to Culebra [48]. As a result, *Culebrenses* could demonstrate a greater inherent consciousness of protecting natural resources, the environment, and fragile ecosystems, which are also the island’s main tourist attractions. The results of this study help to empirically reaffirm Culebra’s positive community attitudes and beliefs toward the environment. Although the intentions to protect the island were in the minds of community members, these intentions did not necessarily translate into the use of ¡mZAP! to protect the environment and address environmental health risks that affect islanders.

This study suggests that less time spent living in Culebra was associated with stronger positive attitudes toward the environment. This finding is important, particularly considering that most ¡mZAP! users have lived in Culebra for >11 years. Perhaps these results suggest that the longer one lives in a location, the more likely one is to be disincentivized to engage in behaviors to respond to community environmental challenges. For example, *Culebrenses* have been exposed to many environmental health risks in the past, including solid waste polluting beaches, improper land development, air pollution, and most notably, the US military bombing practices [35,63-67]. Although the community advocated against these environmental injustices in the past [66,67], the ongoing repercussions of these challenges [31,68] have potentially resulted in reduced enthusiasm. ¡mZAP! users had higher positive attitudes toward the environment, which could also translate into an increase in environmental conscientiousness.

Participants’ beliefs that government agencies are responsible for responding to environmental risks and community discrepancies related to environmental challenges help us better understand *Culebrenses*’ perceptions of lack of control over the community’s health. Societal issues that affect the community’s poor health outcomes extend into the social and ecological determinants of health [69,70]. The ongoing dependency on government-based solutions to community problems is worrisome, especially after the hurricane experiences of 2017 (which may potentially continue to be an issue subsequent to the more recent 2020 earthquakes).

The catastrophic impact of hurricanes Irma and María and the inappropriate responses of the Puerto Rican and US governments may have cemented this perception of lack of control. Community members experienced the isolation of being disconnected from the main island of Puerto Rico and the exacerbation of an unreliable maritime transportation system, resulting in a societal crisis where health access, common goods, and food became scarce [71,72].

*Culebrenses*’ dependency systems and the way social injustices from the past and the present may have resulted in the perception of lack of control, which was associated in this study with negative environmental attitudes, can undermine the good intentions of community members to protect their land. Therefore, implementing community-driven approaches to address environmental health risks or health disparities may not be sufficient to ensure a successful mHealth tool intervention, especially when other social conditions work as oppressors. Socioecological conditions force community priorities to be in constant transformative change. This transformative change was especially true after the 2017 hurricane season in Culebra, where the emergence of new challenges such as rebuilding destroyed properties became a new top priority for *Culebrenses*.

Previous studies suggest that new mobile apps are used between 4% and 20% of the time, with an average session app use of <1 minute. Furthermore, 51% of apps are deleted after the first week of use [73-78]. In this study, only 12.6% (14/111) of participants adopted the use of ¡mZAP!. Although the number of initial users was small, it may be noteworthy that 36% (5/14) of participants used the mHealth tool more than once. These adoption and reuse rates are typical when compared with the adoption rates of other mobile apps. In addition, identifying proper champions on the island to promote ¡mZAP! presented a challenge that could have also affected the use of the mHealth tool. Early identification of community champions has been previously studied as an approach to develop and increase trust between community members and investigators, facilitating an engaging process and a successful community-based research intervention [79-81].

This research study lacked sufficient power to detect some potentially meaningful and theoretically driven associations. The observed associations that were not statistically significant but which had ORs >1.5 can, therefore, be useful for hypothesis generation and to inform future studies. Therefore, in a larger sample, certain relevant predictor variables would have statistically significant associations with the primary outcome, such as age, education level, cell phone number and type of internet per household, daily use of electronic devices, and the perception of how technology contributes to help fixing community problems.

The results of this study provide some support for suggesting that people used ¡mZAP! and were taking self-directed action by using ICTs to address environmental health challenges in Culebra. These results, although limited, confirm the objective of this study to assess the ability of ICTs as tools to engage islanders in collective actions that address environmental health risks. However, for other *Culebrenses*, although they may have similar intentions to protect the environment, existing higher community priorities and potential oppressive challenges, including health care and maritime transportation access, prevent them from translating their intentions into actions.

### Limitations and Strengths

Although this pilot study may offer some insight into directional relationships via statistically significant associations in this sample, interpretation of results is limited because of the small sample size, which restricted power. There may have been a selection bias, including volunteer bias. The data collected for this study reflect a one-time *snapshot* where questions were self-administered by participants, potentially generating respondent bias. The hurricane disasters of 2017 may have substantially affected community perceptions and priorities to the extent that the development of ¡mZAP! was affected in unexpected ways.

The empirical findings concerning perceived lack of control and government dependency systems support conclusions about how these situations could have a stronger influence on people’s behaviors. These behaviors could lead to a perpetuation of the challenges associated with this population’s unique social determinants of health. The results from this study should be further qualified by the fact that people who are less familiar with technology may not be as represented in this study as those who are more familiar (eg, those who are younger and have higher education). These contextual factors are important in future research to ensure the potential adoption and success of any ICT.

The study results might only apply to the users of the ¡mZAP! app, a tool that proactively focuses on environmental health risks. Anecdotally we know that some community members were positively affected by the tool (social influence from other users). The perceptions and priorities of nonusers are likely to differ. Future studies could explore the relationship between offline and internet-based generations to address communities’ environmental priorities.

This study had several strengths. Existing partnerships with collaborators in Puerto Rico facilitated access to the target population and necessary local ethical review and approval. The study addressed key goals and objectives of Healthy People 2030, including “Use health communication strategies and health information technology to improve population health outcomes and health care quality, and to achieve health equity” [82]. This is the first study to provide an overview of *Culebrenses’* intentions to protect the island’s environment in the context of mHealth resource use. In addition, this study provides a unique perspective on how people in remote and underresourced communities perceive environmental health risk and how those perceptions affect the use of rapidly advancing mobile technologies, which can help decrease barriers to access to health in rural areas [2,83,84]. In conclusion, the study findings demonstrated the capacity to stimulate collective action by using ICTs as a novel and engaging approach in underresourced rural locations.

### Implications and Future Research

Future research studies should seek to better understand the factors preventing the use of ¡mZAP! and other ICTs, including changes in the community’s perceptions and priorities after natural disasters or other major community-wide challenges. It is important to explore how ICT use can support, facilitate, or even drive collective community actions. For example, there are multiple community-based centers, groups, and institutions, including a federally qualified health center, a women’s health community organization, and other grassroot-level environmental organizations that can be supplied with ¡mZAP! and other mHealth ICT–related mobile technologies. These tools could be coupled with educational materials and community-based initiatives aimed at increasing positive perceptions of the environment by community members in this rural setting.

Future research should work toward a better understanding of community members’ priorities and addressing pre-existing social determinants, such as those found in this study. Although other social determinants of health in this study, including colonialism, were not assessed, this research establishes a contributing baseline to further investigate the relationship between health or environmental disparities and the sociopolitical power imbalances that affect community islands such as Culebra [85,86]. As community-driven interventions have been successful in the past, with the understanding that community work takes time to develop, the findings of this study can serve as a foundation for future community and ICT research in Culebra and other locations with similar environmental health conditions.

## Abbreviations

¡mZAP!: Zonas, Acción y Protección
a-EAI: adapted environmental attitudes inventory
FAITH!: Fostering African American Improvement in Total Health
ICT: information and communication technology
mHealth: mobile health
MHLC: multidimensional health locus of control
OR: odds ratio
REDCap: Research Electronic Data Capture
SCT: social cognitive theory
